# OCT biomarkers as predictors of visual improvement in diabetic macular edema eyes receiving dexamethasone implants

**DOI:** 10.1186/s40942-023-00473-w

**Published:** 2023-06-14

**Authors:** Giacomo Visioli, Ludovico Alisi, Elvia Mastrogiuseppe, Giuseppe Maria Albanese, Enrico Romano, Ludovico Iannetti, Marta Armentano, Francesca Giovannetti, Magda Gharbiya

**Affiliations:** 1 Department of Sense Organs, Faculty of Medicine and Odontology, Policlinico Umberto I, Sapienza University of Rome, viale del Policlinico 155, Rome, 00161 Italy; 2 Ophthalmology Unit, Head and Neck Department, Policlinico Umberto I University Hospital, Sapienza University of Rome, Rome, Italy

**Keywords:** Diabetic macular edema, Dexamethasone implant, Optical coherence tomography, Subfoveal neuroretinal detachment, Central subfield thickness, Biomarkers

## Abstract

**Background:**

Several optical coherence tomography (OCT) biomarkers have been proposed as predictors for functional and anatomical outcomes in Diabetic Macular Edema (DME). This study aims to examine the impact of these OCT features on the visual acuity improvement of patients with DME after long-acting Dexamethasone intravitreal implants (DEX-I) injection. Furthermore, the safety and impact of DEX-I on clinical parameters, including intraocular pressure (IOP) were assessed.

**Methods:**

In this retrospective observational study, we reviewed the medical records of naïve and non-naïve eyes with DME who received at least one DEX-I. The primary endpoint was visual acuity improvement of ≥ 5 ETDRS letters at 1 month and 4 months after treatment. Secondary outcomes were the changes in OCT biomarkers and the impact of DEX-I on IOP at 1 and 4 months of follow-up. Linear panel regression analysis was used to test for differences in central subfield thickness (CST) over time and it was stratified according to biomarkers at baseline. Finally, a logistic regression analysis was used to identify factors predicting visual improvement at 1 and 4 months.

**Results:**

We included 33 eyes of which 63.6% were at an advanced stage of DME. Overall, CST, cube average thickness (CAT), cube volume (CV), and intraretinal cystoid spaces > 200 μm (ICS) decreased following DEX-I injection (p < 0.001). Additionally, a thicker CST at baseline was observed in eyes with better visual improvement at one month (p = 0.048). After logistic regression analysis, CST was retained as the only predictor for visual improvement at one month (p = 0.044). Furthermore, panel regression analysis identified a relation between subfoveal neuroretinal detachment (SND) at baseline and CST increase at four months. Lastly, only 15.2% of the eyes necessitated topical medication for IOP reduction, with no differences observed when stratifying between naïve and non-naïve eyes.

**Conclusion:**

Our analyses suggest that a ticker baseline CST may serve as a positive predictor of early visual improvement and SND presence at baseline may be a negative prognostic factor for CST increase 4 months after DEX-I injection. Other well-known biomarkers, such as disorganization of the inner retinal layers (DRIL) and hyperreflective foci (HF), did not demonstrate prognostic value on visual outcomes, at least within the first four months following the injection.

**Supplementary Information:**

The online version contains supplementary material available at 10.1186/s40942-023-00473-w.

## Introduction

Diabetes mellitus (DM) is one of the most prominent health issues in the modern world. Nowadays, the world health organization estimates place the number of diabetic people around 442 million. Projections for the future define a strong growing trend [1].

One of the most common complications of DM is diabetic retinopathy (DR). Around 50% of diabetic patients develop DR 10 years after the diagnosis, this percentage rises to 90% 25 years after the diagnosis [2]. DR is one of the main causes of blindness worldwide and is responsible for the severe vision impairment of around 2.6 million people [3].

The most common cause of vision loss in DM patients is diabetic macular edema (DME), conventionally defined as the retinal thickening or the presence of hard exudates within 1 disk diameter of the center of the macula [4]. The prevalence of DME ranges between 2.7 and 11% of the diabetic population [5]. The incidence tends to increase with the disease severity, involving 3% of mild non-proliferative DR and up to 71% of patients with proliferative DR [6]. DME represents the clinical manifestation of the accentuated permeability of the retinal capillaries, the breakdown of the blood-retinal barrier (BRB), and the altered homeostasis of Muller cells that leads to the accumulation of intraretinal fluid [7].

The pathogenesis of DME is complex and only partially related to hyperglycemia. Long-term exposition to hyperglycemia, inflammation and oxidative stress all play a role in the disruption of the BRB [8]. On a molecular level, vascular endothelial growth factor (VEGF), inflammatory chemokines (e.g., CCL2, CCL5, CXCL8), and cytokines (e.g. IL-6, IL-8, IL-1β, and TNF-α), as well as adhesion molecules are all involved in the development of DME [9]. In the early stages of the disease the edema is responsible for the reduced visual acuity through the alteration of the retinal thickness and refractive index. In the later stages of the disease, ischemia and disorganization of the inner retinal layers, caused by glial reaction and neuroretinal damages are the causes of irreversible vision loss [10].

Several approaches have been suggested for the treatment of DME, namely corticosteroids intravitreal or retrobulbar injections, intravitreal anti-VEGF injections, and laser treatments.

As for steroid implants, the currently available molecules are triamcinolone acetonide (TA), fluocinolone acetonide (FA), and dexamethasone. Corticosteroids exert their therapeutic effects through the reduction of VEGF expression, the inhibition of leukostasis and inflammatory molecules, and the reconstitution of the BRB [11].

Dexamethasone is available as an intravitreal implant in Europe and America in sustained-release formulation (Ozurdex TM Allergan Inc., Irvine, California, USA) [12].

Nowadays, steroid therapy is considered a second-line therapy in patients unresponsive to anti-VEGF [13]. Nevertheless, numerous studies have demonstrated the efficacy of Dexamethasone implants in DME. Reports from the MEAD study group found that after 3 years of treatment, dexamethasone determined an improvement of 15 letters or more in 22% of the patients compared to 12% of the patients in the sham group [14]. Despite the efficacy, at the end of the follow-up, in phakic patients, 59.2% of eyes required cataract surgery; 41.5% of eyes required ocular hypotensive therapy [14].

Optical coherence tomography (OCT) is one of the most accurate methods to evaluate the treatment efficacy of intravitreal implants in DME. Numerous OCT biomarkers have been suggested to predict the functional and anatomical outcomes of different treatments. Saxena et al. demonstrated that mean central subfield thickness (CST), cube average thickness (CAT), and cube volume (CV) are all independent markers of DME severity and prognostic factors for visual acuity [15]. Subfoveal neuroretinal detachment (SND) at baseline was associated with a better functional outcome after the Dexamethasone implant [16]. The presence and size of intraretinal cystoid spaces (ICS) within the macula has been also suggested as a biomarker of visual outcome in several studies [17]. Similarly, the presence and size, and localization of hyperreflective foci (HF) within the retina may have a similar prognostic value [18]. The disorganization of the inner retinal layers (DRIL) was found negatively correlated with the functional outcome of DME [19]. The presence of vitreomacular traction is a common recurrence in diabetic patients due to the tout posterior hyaloid and it’s believed to cause recalcitrant macular edema [18]. Lastly, the loss of integrity of the outer retinal layers, specifically the external zone/external limiting membrane (EZ/ELM) has been linked to the accumulation of subretinal edema [20].

The aim of the current study is to observe and summarize the impact of all the aforementioned OCT characteristics on the visual acuity of DME patients. Moreover, we evaluated the impact of intravitreal implants of Dexamethasone on clinical parameters such as intraocular pressure.

## Materials and methods

This retrospective observational study was performed according to the tenets of the Declaration of Helsinki. Informed consent was obtained from all subjects involved in the study. An analysis of clinical records from the Ophthalmology Clinic of the Umberto I Hospital, Sapienza - University of Rome, was performed from March 2020 to September 2022. Inclusion criteria were: age ≥ 40 years old, type 2 diabetes mellitus, DME, history of at least 1 dexamethasone implant (DEX-I), and at least 4 months of follow-up. We included both naïve and treated eyes. Naïve and treated eyes affected by DME were defined as follows: naïve eyes never received any intravitreal injection prior to the dexamethasone implant (DEX-I) injection; treated eyes previously underwent anti-vascular endothelial growth factor (anti-VEGF) intravitreal injection but had not received any previous DEX-I injections.

Patients who had a history of retinal vein occlusion, retinal detachment, uveitis, neovascular age-related macular degeneration or choroidal neovascularization, visual loss due to other reasons than DME, and recent cataract surgery within the previous 3 months, were excluded. Patients who underwent pars plana vitrectomy were not included. We also excluded patients with low-quality or unreliable OCT scans where biomarkers could not be clearly identified. In the case of bilateral DME and both eyes treated with DEX-I only one eye was randomly included.

As a part of the standardized protocol, every patient included in this study underwent detailed ophthalmologic examination, including BCVA using ETDRS charts, slit lamp biomicroscopy, intraocular pressure (IOP) measured by applanation tonometer, fundus examination, and OCT.

Personal or family medical history suggestive of glaucoma or ocular hypertension was noted. Follow-up visits at one week, one month, and four months after the DEX-I injection were documented. All injections were performed in the operating room following the current guidelines for intravitreal injections.

The OCT scans were obtained using SD-OCT (Spectral Domain, Heidelberg Engineering, Germany) at 1 and 4 months of follow-up.

OCT characteristics were measured by a single experienced ophthalmologist. CST (µm) was calculated as the thickness of the central 1 mm circle in the ETDRS Grid. CAT (µm) was calculated as the mean value of the 9 scans of the 3 × 3 grid, values were extrapolated by automatic segmentation between the retinal pigmented epithelium (RPE) and the inner limiting membrane (ILM). CV (mm3) was calculated as the mean cube volume in the 3 × 3 Grid area between the RPE and the ILM. SND, ICS, DRIL, EZ/ELM alteration, and VMT were highlighted as binomial variables (present/absent). HF were considered as present when they were more than 30 in number [21]. The ICS dimensions were measured using the caliber tool provided with the OCT software. Large cysts were defined by the longest diameter being > 200 microns. Cyst diameter was evaluated in the whole 3 × 3 scan previously employed for the CAT and CV. DRIL was considered present when the boundaries of the layers between the ganglion cells layer and the internal plexiform layer could not be defined. Moreover, DRIL was considered present if consistently found in the foveal scan and in the 3 scans above and below. SHFs, defined as small spots (< 30 microns) with reflectivity similar to the nerve fiber layer and no back shadowing, were evaluated in a similar fashion to DRIL in the same 7 OCT scans. SHFs were considered present if located in the 1 mm area of the ETDRS Grid. EZ/ELM alteration was considered present if, in the central scan, we observed an interruption of any dimension of the external layers. VMT was considered present if the central scan showed any distortion relatable to the traction determined by the posterior hyaloid.

We considered as the main end-point visual acuity improvement at 1 month of at least 5 ETDRS letters. Secondary outcomes were the reduction of macular edema evaluated through CST, CAT, and CV parameters, and the changes in the binomial parameters (SND, ICS, DRIL, SHF, HF, EZ/ELM alteration and VMT) at 1 and 4 months of follow-up. Moreover, we classified the DME, according to the ESASO (European School for Advanced Studies in Ophthalmology) classification. Patients were stratified into early, advanced, severe, and atrophic maculopathy based on the presence or the staging of retinal thickness, cysts, EZ interruption, and DRIL [21].

### Statistical analysis

Graph generation and statistical analysis were carried out using STATA, v. 17.0 (StataCorp, TX, USA). Continuous variables, reported as mean ± standard deviation (SD), were tested for normal distribution by the Shapiro–Wilk test. To compare non-parametric values the Mann–Whitney test was employed, whereas the unpaired t-test was used to compare parametric values. The Pearson coefficient or Spearman’s rank correlation was employed accordingly to evaluate bivariate correlations. Categorical variables were reported as counts and percentages and were compared with Fisher’s exact test. A linear panel regression analysis reporting marginal effects was run to test for the differences of CST over time stratified by the OCT biomarkers SND, DRIL, SHF, and EZ/ELM alteration along the follow-up points (0, 1, and 4 months). A post-hoc sample size evaluation has been performed using G*Power 3.1.9.6 computing the differences between the mean CST values among the group with a visual improvement ≥ 5 ETDRS and the group with a visual improvement < 5 letters at one month [22]. Input data were as follows: two tails and α 0.05. The effect size was calculated using mean CST and SD of each group.

To identify the factors predicting a visual improvement ≥ 5 ETDRS letters at one month, a stepwise logistic regression analysis corrected by age and sex was assessed. A similar model was run for assessing factors predicting visual improvement ≥ 5 ETDRS at 4 months. Factors associated with visual improvement at one month in the bivariate relationships (p < 0.05) together with relevant clinical factors (lens status, IOP, spherical equivalent, pre-DEX-I visual acuity) were included in the model. Factors with p < 0.05 after the logistic regression were retained as final predictors for visual improvement. When appropriate, we reported confidence intervals (CI 95%) and p-values.

## Results

**Fig. 1:**
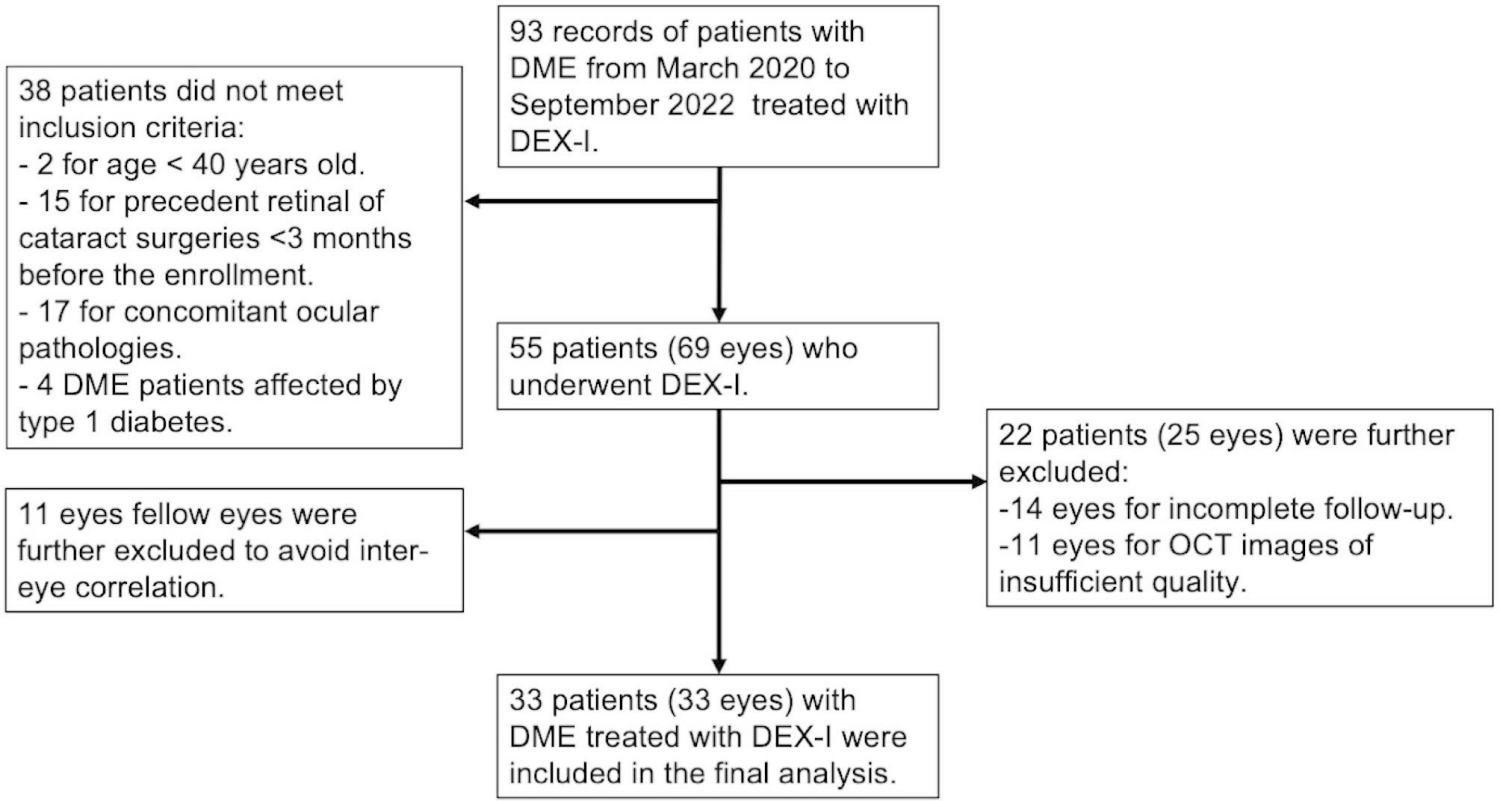
Flow chart of the retrospective study: after the application of a strict protocol of inclusion and exclusion criteria, 33 eyes with diabetic macular edema (DME) treated with dexamethasone implant (DEX-I) were included. OCT: optical coherence tomography

For the present study, we retrieved the clinical records of 93 patients. After the application of the inclusion and exclusion criteria, we selected 69 eyes from 55 patients. After OCT analysis we further excluded 22 patients (25 eyes) for incomplete follow-up or low-quality OCT images. For patients who were treated in both eyes, we randomly selected only one eye and for this reason, we excluded 11 eyes. Finally, we included 33 eyes of 33 patients in the statistical analysis (Fig. 1). Of those, 18 patients (54.5%) showed an improvement greater than or equal to 5 letters in ETDRS chart at one month. Overall, we included 5 eyes with early DME, 21 with advanced DME, 5 with severe DME, and 2 patients with atrophic maculopathy. Table 1 summarizes the main demographic and clinical characteristics of the studied population stratified for the EDTRS improvement (< or ≥ than 5 letters) at 1 and 4 months of follow-up. Based on the mean CST values and SD at 1 month between the two groups, the effect size was set at 0.73. The output parameters evaluating the statistical power were as follows: non-centrality parameter δ = 2.089, critical t = 2.039, DF = 31, power = 0.53. Demographic characteristics showed that 15 (45.4%) patients were female and 18 (54.6%) were male. The mean age was 68.2 ± 9.7. Out of all the participants, 15 (45.4%) had undergone cataract surgery at the time of enrollment, and none of the patient showed more than a mild cataract during the entire observation period. No consistent differences were observed in the demographic and functional characteristics at baseline between the patients that achieved an improvement of at least 5 letters, and the patients that showed an improvement of less than five letters both at 1 month and 4 months (Table 1).

**Table 1:** Demographic and clinical characteristics of the observed population at baseline. Results are stratified by letter improvement 1 month and 4 months after the dexamethasone implant

	Total	ETDRS improvement (1 month)			ETDRS improvement (4 months)		
	n = 33	< 5 letters (n = 15)	≥ 5 letters (n = 18)	*P value*	< 5 letters (n = 17)	≥ 5 letters (n = 16)	*P value*
Sex (female), n (%)	15 (45.4%)	8 (53.3%)	11 (61.1%)	0.54*	10 (58.8%)	9 (56.3%)	1.00*
Age, mean ± SD	68.2 ± 9.7	67.6 ± 12.4	68.6 ± 7.2	0.96°	67.6 ± 11.6	68.8 ± 7.6	0.93°
ETDRS letters, mean ± SD	28.7 ± 16.0	31.9 ± 17.8	26.1 ± 14.3	0.29°	32.5 ± 18.5	25.3 ± 12.4	0.14°
LogMar, mean ± SD	0.59 ± 0.46	0.53 ± 0.50	0.64 ± 0.43	0.26°	0.56 ± 0.57	0.62 ± 0.31	0.15°
SEQ, mean ± SD	0.36 ± 1.14	0.38 ± 1.15	0.33 ± 1.16	0.90°	0.46 ± 1.18	0.25 ± 1.13	0.61°
Pseudophakia, n (%)	15 (45.4%)	5 (33.3%)	10 (55.5%)	0.30*	8 (47.1%)	7 (43.8%)	1.00*
Naïve, n (%)	21 (63.6%)	10 (66.7%)	11 (61.1%)	1.00*	10 (58.8%)	11 (68.8%)	0.72*
Laser treatment, n (%)	14 (42.4%)	9 (60.0%)	5 (27.8%)	0.09*	8 (47.1%)	6 (37.5%)	0.73*

ETDRS: Early Treatment of Diabetic Retinopathy Study; SD: stendard deviation, SEQ: spherical equivalent. *Fischer’s exact test °Unpaired t-test

The analysis of volumetric and morphological characteristics at baseline is shown in Table 2. Baseline measurements are stratified by letter improvement < or ≥ 5 letters at 1 and 4 months. The mean CST at baseline was 487.0 ± 142.0 μm and when stratifying data, patients who showed an improvement ≥ 5 letters at 1 month had ticker CST at enrollment (531.2 ± 153.9 μm p = 0.048). No other relevant differences were highlighted when comparing the other retinal biomarkers. However, it should be noted that CAT and CV at baseline, similarly as observed for CST, tended to be higher – even if not significantly – in patients with better visual outcomes at 1 month (p = 0.138 and p = 0.143, respectively).

**Table 2:** OCT morphological characteristics of the observed populations at baseline. Results are stratified by letter improvement 1 month and 4 months after the dexamethasone implant

	Total	ETDRS improvement (1 month)			ETDRS improvement (4 months)		
	n = 33	< 5 letters (n = 15)	≥ 5 letters (n = 18)	*P value*	< 5 letters (n = 17)	≥ 5 letters (n = 16)	*P value*
CST µm, mean ± SD	487.0 ± 142.0	433.9 ± 108.6	531.2 ± 153.9	**0.048**°	487.4 ± 168.9	486.6 ± 112.1	0.980°
CAT-3 mm µm, mean ± SD	448.6 ± 97.7	413.2 ± 61.2	478.1 ± 113.2	0.138°	454.9 ± 118.3	441.9 ± 73.0	0.885°
CV-3 mm mm^3^, mean ± SD	3.17 ± 0.72	2.91 ± 0.42	3.39 ± 0.86	0.143°	3.20 ± 0.81	3.14 ± 0.64	0.943°
SND, n (%)	9 (27.3%)	2 (13.3%)	7 (38.9%)	0.134*	2 (11.8%)	7 (43.7%)	0.057*
ICS > 200 μm, n (%)	32 (96.9%)	15 (100%)	17 (94.4%)	1.000*	17 (100%)	15 (93.7%)	0.485*
DRIL, n (%)	10 (30.3%)	4 (26.7%)	6 (33.3%)	0.722*	6 (35.3%)	4 (25.0%)	0.397
SHF, n (%)	19 (57.6%)	7 (46.7%)	12 (66.7%)	0.304*	8 (47.0%)	11 (68.7%)	0.296*
HF, n (%)	28 (84.8%)	13 (86.7%)	15 (83.3%)	1.000*	15 (88.2%)	13 (81.2%)	0.656*
EZ/ELM alteration, n (%)	19 (57.6%)	8 (53.3%)	11 (61.1%)	0.733*	11 (61.1%)	8 (50.0%)	0.491*
VMT, n (%)	6 (18.2%)	3 (20.0%)	3 (16.7%)	1.000*	3 (16.7%)	3 (18.7%)	1.00*
IOP, n (%)	14.8 ± 2.0	14.7 ± 1.8	14.8 ± 2.2	0.951*	14.8 ± 1.1	14.7 ± 2.7	0.850*

CAT: cube average thickness, CST: central subfoveal thickness, CV: cube volume, DRIL: disorganization of the inner retinal layers, EZ/ELM: ellipsoid zone/ external limiting membrane, ICS: Intraretinal Cystoid Spaces, IOP: intraocular pressure, HF: hyperreflective foci, SHF: Subfoveal hyperreflective foci, SND: Subfoveal Neuroretinal Detachment, VMT: vitreomacular traction. *Fischer’s exact test °Unpaired t-test

**Fig. 2:**
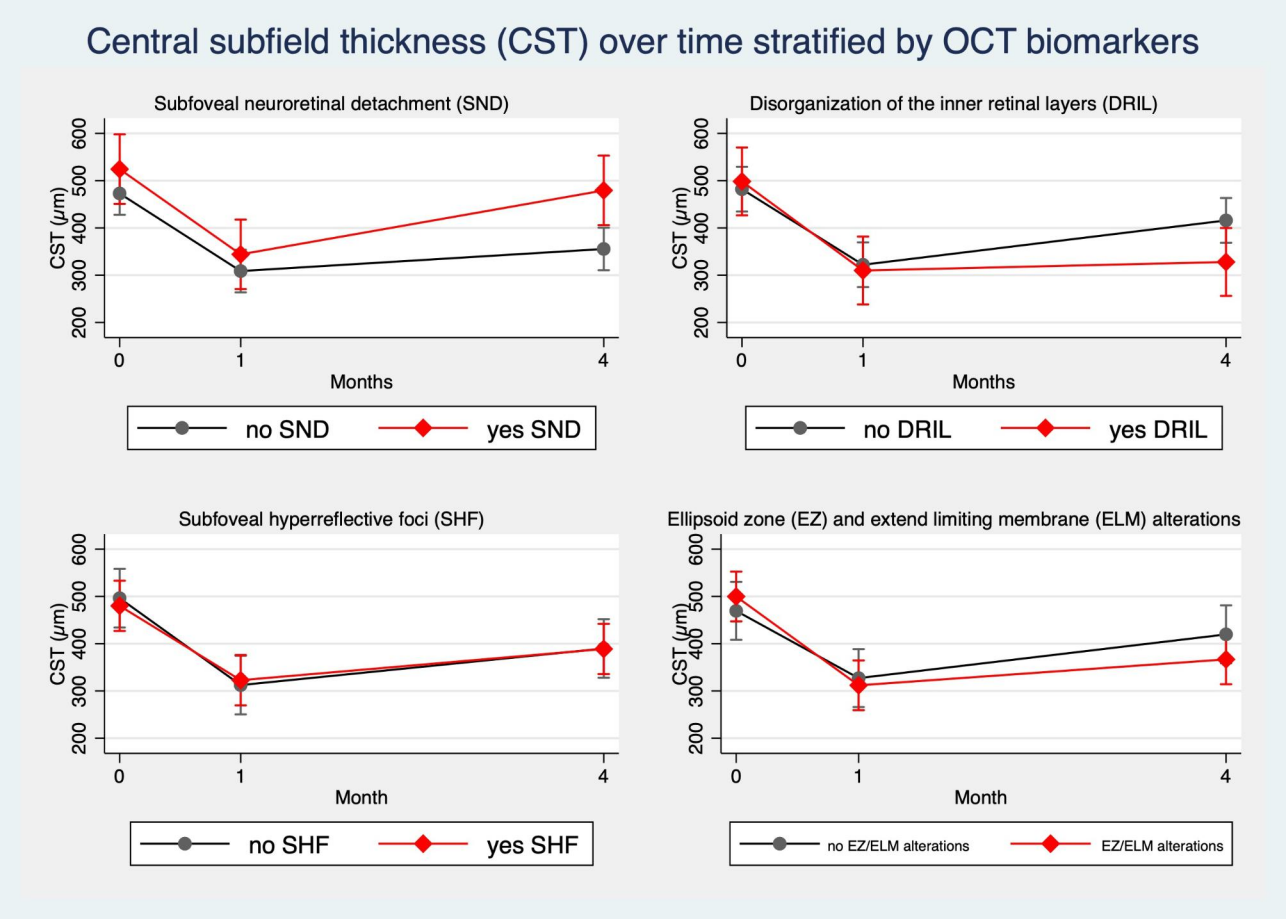
Margins plot of central subfield thickness (CST) over time stratified by four OCT biomarkers: subfoveal neuroretinal detachment (SND), disorganization of the inner retinal layers (DRIL), subfoveal hyperreflective foci (SHF) and External Zone/External Limiting Membrane alterations (EZ/ELM)

Table 3 shows OCT biomarkers at baseline and at every point in the follow-up. CST, CAT, and CV were consistently lower at 1 and 4 months compared to baseline. Comparing these factors with Tukey post-hoc only CST showed an increase between 1 and 4 months (contrast 70.88 ± 28.70, p = 0.04), while CAT and CV did not substantially differ. Regarding the other biomarkers studied, no other differences were observed except from large ICS (> 200 microns) which exhibited a consistent decrease from 32 patients at baseline to 19 at 1 month and 22 at 4 months (p < 0.001). Additionally, we evaluated the same biomarkers at both 1 month and 4 months, stratifying the included eyes into naïve and treated eyes. As shown in Table 4, we did not find any significant differences between naïve and treated eyes in their response to DEX-I treatment. The potential influence of the morphological binomial parameters before treatment on the CST during the follow-up has been evaluated through a panel regression analysis. As a result of the model, we identified SND as a potential negative prognostic factor for CST increase at 4 months (Fig. 2).

**Table 3:** OCT biomarkers of diabetic macular edema (DME) eyes at baseline and at 1 month and 4 months after dexamethasone implant

	Baseline (n = 33)	1 month (n = 33)	4 months (n = 33)	p-value
CST µm, mean ± SD	487.0 ± 142.0	318.4 ± 61.7	389.3 ± 129.6	**< 0.001°**
CAT-3 mm µm, mean ± SD	448.6 ± 97.7	357.3 ± 42.2	378.5 ± 99.1	**< 0.001°**
CV-3 mm mm3, mean ± SD	3.17 ± 0.72	2.53 ± 0.30	2.73 ± 0.53	**< 0.001°**
SND, n (%)	9 (27.2%)	4 (12.1%)	8 (24.2%)	0.298*
ICS > 200 μm, n (%)	32 (96.9%)	19 (57.6%)	22 (66.6%)	< 0.001*
DRIL, n (%)	10 (30.3%)	14 (42.4%)	9 (27.2%)	0.492*
SHF, n (%)	19 (57.6%)	21 (63.6%)	22 (66.6%)	0.811*
HF, n (%)	28 (84.8%)	30 (90.9%)	27 (81.8%)	0.572*
EZ/ELM alteration, n (%)	19 (57.6%)	23 (69.7%)	17 (51.5%)	0.359*
VMT, n (%)	6 (18.2%)	7 (21.2%)	9(27.2%)	0.755*

CAT: cube average thickness, CST: central subfoveal thickness, CV: cube volume, DRIL: disorganization of the inner retinal layers, EZ/ELM: ellipsoid zone/ external limiting membrane, ICS: Intraretinal Cystoid Spaces, IOP: intraocular pressure, HF: hyperreflective foci, SHF: Subfoveal hyperreflective foci, SND: Subfoveal neuroretinal detachment, VMT: vitreomacular traction. *Fischer’s exact test. °Unpaired t-test

**Table 4:** Functional and structural outcomes in naïve and non-naïve (treated) eyes at 1 and 4 months

	1 month (n = 33)		p-value	4 months (n = 33)		p-value
	Naïve eyes (21)	Treated eyes (12)		Naïve eyes (21)	Treated eyes. (12)	
CST µm, mean ± SD	331.9 ± 57.6	294.8 ± 64.0	0.098°	409.5 ± 133.7	354.0 ± 119.3	0.243°
CAT-3 mm µm, mean ± SD	358.8 ± 38.8	356.4 ± 44.9	0.877°	371.5 ± 115.3	390.6 ± 64.7	0.603°
CV-3 mm mm3, mean ± SD	2.54 ± 0.30	2.53 ± 0.30	0.985°	2.72 ± 0.59	2.75 ± 0.45	0.888°
SND, n (%)	2 (9.5%)	2 (16.7%)	0.610*	4 (19.1%)	4 (33.3%)	0.420*
ICS > 200 μm	20 (95.2%)	8 (66.7%)	0.047*	19 (90.5%)	10 (83.3%)	0.610*
DRIL, n (%)	11 (52.4%)	3 (25.0%)	0.160*	7 (33.3%)	2 (16.7%)	0.429*
SHF, n (%)	14 (66.7%)	7 (58.3%)	0.716*	15 (71.4%)	7 (58.3%)	0.471*
HF, n (%)	20 (95.2%)	10 (83.3%)	0.538*	18 (85.7%)	9 (75.0%)	0.643*
EZ/ELM alteration, n (%)	15 (71.4%)	8 (66.7%)	1.000*	11 (52.4%)	6 (50.0%)	1.000*
VMT, n (%)	3 (14.3%)	4 (33.3%)	0.377*	7 (33.3%)	2 (16.7%)	0.429*

CAT: cube average thickness, CST: central subfoveal thickness, CV: cube volume, DRIL: disorganization of the inner retinal layers, EZ/ELM: ellipsoid zone/ external limiting membrane, ICS: Intraretinal Cystoid Spaces, IOP: intraocular pressure, HF: hyperreflective foci, SHF: Subfoveal hyperreflective foci, SND: Subfoveal neuroretinal detachment, VMT: vitreomacular traction. *Fischer’s exact test °Unpaired t-test

**Fig. 3:**
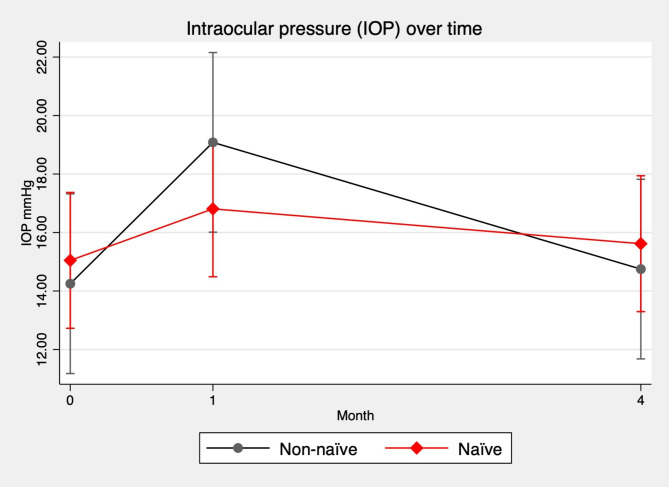
Margins plot of intraocular pressure (IOP) over time in patients who underwent dexamethasone implant (DEX-I), stratified by naïve and treated eyes (non-naïve)

To identify final predictors for ETDRS improvement ≥ 5 letters at one month, we run the stepwise logistic regression analysis (cons. -3.93, CI: -7.72 to -0.12, pseudo R2 = 0.14, p = 0.043) and thicker CST was retained as the unique predictor for visual improvement at 1 month (coeff. 0.01, CI 0.00 to 0.01, p = 0.044). We performed an analogous model to assess visual improvement at 4 months that did not highlight any predictive factor (cons. 0.91, CI: -1.56 to 3.38, pseudo R2 = 0.16 p = 0.468). Safety analysis revealed no major complications such as endophthalmitis or insert dislocation to the anterior chamber. No patient showed signs of glaucoma before the DEX-I. Five (15.15%) patients showed elevated intraocular pressure (IOP) at the 1-month follow-up that required topical medications. In Fig. 3 the trend of IOP elevation is shown with a peak at 1-month (mean 17.6 ± 8.3) with no substantial differences when stratified by naïve and non-naïve (treated) eyes. Overall, 4 patients (12.1%) of patients showed an elevated IOP that required topical therapy. At the 4-month follow up the number of patients that required topical medications due to elevated IOP was 3 (9.1%).

## Discussion

The main objective of this retrospective observational study was to assess the influence of different baseline OCT characteristics on the visual acuity of patients with DME during the initial months following DEX-I treatment. Our results suggest that thicker CST at baseline may be a predictor for visual improvement at one month, and subfoveal neuroretinal detachment SND may be a negative prognostic factor for CST increase at 4 months.

In the literature, the efficacy of DEX-I has been investigated and has been widely compared to anti-VEGF which is currently considered the gold standard for DME [23]. The principal limitations of employing DEX-I are a higher incidence of IOP elevation and cataract formation compared to anti-VEGF. However, the role of corticosteroids, particularly DEX-I, in the management of DME remains an active area of research. A meta-analysis by He et al. compared the efficacy of DEX-I versus anti-VEGF in DME and found that both treatments were effective in improving visual acuity [13]. The authors concluded that despite DEX-I having relatively superior anatomic outcomes compared with anti-VEGF, due to the higher risk of IOP elevation and cataract progression, it may be considered first-line therapy for DME especially in select cases like pseudophakic patients or anti-VEGF-resistant eyes. Furthermore, a recent study highlighted that a lower number of dexamethasone intravitreal (DEX-I) injections offers an advantage compared to anti-VEGF treatments, particularly in pseudophakic patients [24].

Regarding our principal finding, the association between CST and visual outcomes is consistent with the previous literature. In this setting, some studies have already considered the macular thickness and volumetric parameters in patients with DME as they are easy to determine and require no further processing of the images. Saxena et al. demonstrated that CST, CAT, and CV were all independent markers of the severity of diabetic retinopathy and prognosticators of visual acuity [15]. Conversely, Valentim et al. conducted a post-hoc analysis which revealed that higher CST may have a significant impact on the time required for DME resolution, as it was found to be associated with a longer median time of edema reduction [25]. However, a greater CST may also indicate an early or advanced stage of diabetic macular edema, with a greater likelihood of morphological and functional recovery. The ESASO classification reflects this by considering an increase in CST of less than 10% above upper normal values as a principal negative prognostic factor, indicating the stage of atrophic maculopathy [21].

Regarding SND, this biomarker ranges between 15% and 30% in different reports [26]. Our results align with the literature as SND was seen in 27.2% of the patients. The presence of SND has been reported both as a positive and negative predictive factor for functional outcomes in various studies. Seo et al. reported a more frequent disruption of the photoreceptors and a worse visual outcome in patients with concomitant SND. Moreover, they observed that SND is often associated with ELM and RPE disruption and tends to answer poorly to anti-VEGF [27]. Similarly, Giocanti-Aurégan et al. reported that the presence of subretinal fluid did not significantly influence the VA improvement after intravitreal injection of anti-VEGF [28]. Vujosevic et al. found that the presence of SND determined a higher central thickness, a disruption of the ELM, and reduced retinal sensitivity [26].

On the other hand, Bonfiglio et al. found that in DME patients that responded poorly to ranibizumab intravitreal injections, those who presented an SND tended to have a better functional and anatomical response than those without SND [29]. Other studies demonstrated that SND may act as a predictive factor only for the anatomical response to therapy [30]. It is worth noting that DME associated with SND and HRS is a unique inflammatory pattern that may respond better to treatment with dexamethasone rather than with intravitreal injections of ranibizumab [31]. Moon et al. reported a better anatomical result (as central retinal thickness) at 3 months after DEX-I in patients with SND when compared to other patterns of DME [16].

The exact pathogenesis of SND is still debated, although it is generally considered to be associated with compromised integrity of the ELM. For example, Otani et al. have suggested that SND is a result of ELM disruption, jeopardizing functional outcomes [32]. One of the most prominent hypotheses is that SND begins with the extravasation of lipids and proteins from the retinal circulation, leading to the development of retinal edema. Subsequently, the loss of integrity of the ELM may allow the edema to accumulate in the subretinal space, exceeding the absorption capacity of the RPE and causing the SND [26]. In the current study, the presence of SND at baseline was found to be a negative predictor for central subfield thickness (CST) at 4 months. This finding is consistent with the described structural compromission determined by SND, especially in chronic ocular diseases. However, further studies are needed to clarify the exact implications of SND on visual outcomes and to identify effective treatments for this condition.

Regarding the remaining biomarkers examined in this study, including ICS, DRIL, SHF, HF, EZ/ELM alteration, and VMT, we did not observe any significant association with visual improvement at either 1 or 4 months, even after stratifying by naïve and treated eyes. While these findings may seem inconsistent with previous literature, it is possible that a longer follow-up period (more than four months) may have yielded more informative results for these biomarkers. For example, Vadalà et al. found that the reduction of HF at 12 and 24 months was correlated with a visual acuity improvement after DEX-I treatment [33]. Moreover, Schreur et al. demonstrated that a higher HF count at baseline is associated with improved visual outcomes following anti-VEGF therapy [34]. However, as highlighted in our study, this relationship may not hold true for DEX-I injections. Similarly, patients without DRIL at baseline tend to have more favorable anatomical outcomes [35]. However, in our study focusing on the relationship between the absence of DRIL and early visual outcomes, we were unable to establish the prognostic role of DRIL. Therefore, further studies with extended follow-up periods are required to investigate the long-term predictive value of these biomarkers especially on visual acuity outcomes.

Regarding safety, our study found no major complications such as endophthalmitis or insert dislocation to the anterior chamber. However, 15.15% of patients showed elevated IOP at the 1-month follow-up. These results are consistent with previous studies reporting elevated IOP as a potential side effect of DEX-I injections. Therefore, patients who receive DEX-I injections should be closely monitored for IOP changes. Despite a rigorous application of inclusion and exclusion criteria, accurate characterization and classification of included eyes as well as OCT biomarkers, the stratification between naïve and non-naïve eyes, the shortcomings of our study should be disclosed. In fact, the retrospective design, the small sample size as well as a suboptimal test power limit the generalizability of our findings. Further, the distribution of our sample population was not homogeneous. Indeed, as shown by the ESASO classification [21], most of our patients fell in the advanced DME group.

## Conclusions

In conclusion, our study suggests that baseline CST may be a predictor for visual improvement at one month, and SND may be a negative prognostic factor for CST increase at 4 months. Our findings support the use of OCT biomarkers to predict treatment response and monitor disease progression in DME patients receiving DEX-I injections. Further studies are warranted to better elucidate the impact and predictivity of the aforementioned characteristics in the progression and outcome of DME.

## Electronic supplementary material

Below is the link to the electronic supplementary material.

Supplementary Material 1

Supplementary Material 2

## Data Availability

Raw data is not publicly available due to informed consent restrictions, but it is available to researchers upon reasonable request to the corresponding author.

## List of abbreviations

BRB: Blood-Retinal Barrier
CAT: Cube Average Thickness
CCL2: Chemokine (C-C motif) ligand 2
CCL5: Chemokine (C-C motif) ligand 5
CV: Cube Volume
CXCL8: Chemokine (C-X-C motif) ligand 8
CST: Central Subfield Thickness
DM: Diabetes Mellitus
DME: Diabetic Macular Edema
DR: Diabetic Retinopathy
DRIL: Disorganization of the Inner Retinal Layers
ESASO: European School for Advanced Studies in Ophthalmology
EZ/ELM: External Zone/External Limiting Membrane
FA: Fluocinolone Acetonide
HF: Hyperreflective Foci
ICS: Intraretinal Cystoid Spaces
IL: Interleukin
OCT: Optical Coherence Tomography
SHF: Subfoveal Hyperreflective Foci
SND: Subfoveal Neuroretinal Detachment
TA: Triamcinolone Acetonide
TNF: Tumor Necrosis Factor
VEGF: Vascular Endothelial Growth Factor
