# Correction: OCT biomarkers as predictors of visual improvement in diabetic macular edema eyes receiving dexamethasone implants

**DOI:** 10.1186/s40942-025-00644-x

**Published:** 2025-03-04

**Authors:** Giacomo Visioli, Ludovico Alisi, Elvia Mastrogiuseppe, Giuseppe Maria Albanese, Enrico Romano, Ludovico Iannetti, Marta Armentano, Francesca Giovannetti, Magda Gharbiya

**Affiliations:** 1 Department of Sense Organs, Faculty of Medicine and Odontology, Policlinico Umberto I, Sapienza University of Rome, viale del Policlinico 155, Rome, 00161 Italy; 2 Ophthalmology Unit, Head and Neck Department, Policlinico Umberto I University Hospital, Sapienza University of Rome, Rome, Italy

**Correction:** ***Int J Retin Vitr*** **9, 35 (2023)**

10.1186/s40942-023-00473-w

Following the publication of the original article [1], the authors identified a typesetting error in which references 22 and 24 were inadvertently merged, causing both to be unrecognized as separate citations. As a result, all subsequent references were incorrectly numbered.

Reference 24 has been reinstated, and reference 22 has also been corrected.

The original article [1] has been corrected.
