# Supplementary material for: OCT biomarkers as predictors of visual improvement in diabetic macular edema eyes receiving dexamethasone implants
Source: Int J Retina Vitreous. 2023 Jun 14;9:35. doi: 10.1186/s40942-023-00473-w (PMC10265769; doi:10.1186/s40942-023-00473-w)
Supplement: Supplementary file 1 — Supplementary Material 1 [file 40942_2023_473_MOESM1_ESM.docx]

**OCT Biomarkers as Predictors of Visual Improvement in Diabetic Macular Edema Eyes Receiving Dexamethasone Implants**

**Response to reviewers**

**EIC:** We have received strikingly opposed opinions from the reviewers about this manuscript. At this moment, there is no warranty on publication. It will really depend on the authors' answers (point-to-point) and, consequently, on the improved final version of the manuscript (also provide a tracked changes version).

A: Dear editor-in-chief thank you for considering our article. We improved our manuscript following reviewers’ suggestions. As you requested, we have also attached a tracked change version of the manuscript.


**Reviewer #1:** Many thanks for having me look at this interesting manuscript assessing biomarker effectivity in DME treatment while being treated with DeX implant. Though manuscript is well written, it presents limited new information. The discussion can be more concise.

A: Dear reviewer, thank you for evaluating our article. While we acknowledge that our study may not have presented groundbreaking findings, we believe that can contribute to the existing body of knowledge on DME treatment with the DeX implant. By specifically focusing on the improvement of visual acuity and stratifying the population based on a minimum 5-letter improvement, we aimed to provide a more precise functional interpretation of the OCT biomarkers that have already been highlighted in the literature. Based on your report and the reports of the other reviewers, we have made efforts to implement the discussion section.

**Reviewer #2:** I really appreciate your efforts on the processing your manuscript concerning "OCT Biomarkers as Predictors of Visual Improvement in Diabetic Macular Edema Eyes Receiving Dexamethasone Implants".
It is accurate and consistent with the literature and in my opinion, they should be published.
However, I suggest the authors to evaluate the sample size because it is really important when sample is not great. They should use the same methodology adopted in many articles like "De Bernardo M, Altieri V, Coppola A, Gioia M, Rosa N. Choroidal evaluation in patients under alpha-lytic therapy. Graefes Arch Clin Exp Ophthalmol. 2020 Dec;258(12):2729-2736. doi: 10.1007/s00417-020-04907-1. Epub 2020 Sep 2. PMID: 32875394; PMCID: PMC7677155."

A: Thank you for your valuable suggestions. Due to the retrospective nature of our study, we were limited to conducting a post-hoc evaluation of our sample size. Since our objective was to determine the differences between the group with a visual improvement of more than 5 ETDRS letters and the group that did not show such improvement, we were unable to find a reliable “effect size” for this outcome in the existing literature. As a result, the effect size was calculated post-hoc. We acknowledge that the final actual power is not optimal, and we have duly highlighted this limitation in the shortcomings section of our manuscript. We hope that you will find our improvements accurate.

**Reviewer #3:** The authors' reported the results of their experience in treating diabetic maculare edema with dexamethasone implant as far as some commonly known OCT biomarkers.
The author' aim was to investigate the correlation between visual outcomes and these biomarkers in a short term follow-up. The study population is composed by 33 eyes and several parameters were properly considered.
The paper is well constructed, methods are clearly exposed also in definitions and term and in the statistic evaluation, in consideration also of the entity of cohort.
Demonstration of this correlation in such study is however not wide as in authors' purpose and is limited to few of the studied parameters. This should be better underlined in discussion and probably is related to the dicothomic evaluation of most of the parameters also, since it is in some cases in opposition to the current literature. The authors should better clarify their intention considering the results they obtained.

A: Thank you for evaluating our article. We appreciate your feedback and understand your point. We improved the discussions section and we have emphasized the relevance of our findings in relation to previous studies. Additionally, we have highlighted that while most biomarkers have been studied in relation to anatomical outcomes or in the context of anti-VEGF injections, our study focuses specifically on evaluating visual improvement.

Results are of interest in this field because of the need of better understand how to customise treatment. Tables are clear and useful.
Few specific comments:
- pages are not numbered

A: We added the page number
- page 6 row 30: it should be clarified if previous DEXI treated eyes were included in the "treated group"

A: We clarified that the treated eyes were treated with anti-VEGF and never received DEX-I.
- page 6 row 34: "due to other reasons" is apparently out of context

A: Thank you. It was a typo. The complete sentence is “visual loss due to other reasons than DME”.
- vitreoretinal surgery is not an exclusion criteria if performed more than three months before the enrollment: it should be specified in the results how many vitrectomized eyes were included in the cohort because they are considered as a well defined category of eyes with specific responses to treatments.

A: Actually, none of our patients underwent vitrectomy before or during our study. Thank you for bringing up this point, as we have explicitly mentioned it in the methods. Additionally, patients with a history of retinal detachment were not included in our study.
- page 6 line 36: please better define what is "low quality OCT scans"

A: We added “where biomarkers could not be clearly identified”
- page 6 row 58: SHR and HR can't be evaluated as "present or absent" because their value as biomarker is related to quantification. Also in the cited ESASO study there is a "semi-quantification" of this biomarker.

A: Yes. Actually, we did not specify that we considered SHR and HR as present when there were more than 30 in number. We clarified this misunderstanding in the methods section.
- page 8 row 13-14: please better evaluated the sentence (the concept and the expression)

A: We tried to rephrase the sentence
- page 8 row 17: "fine" or "five"?

A: “Five”.
- page 8 row 32: please better specify that the data are not statistically significant

A: Consistent with the existing literature, we would have preferred to avoid using the term "statistically significant." (https://www.nature.com/articles/d41586-019-00857-9). However, in this case, we followed your suggestion as it could assist the reader in quickly interpreting the data.

- page 8 row 40: rephrase

A: Done
- page 8 row 46-49: what does it mean? what is the "subsequent analysis"?

A: We rephrased the sentence
- page 9 row 30: authors of citation 13 concluded their paper differently of what is here reported

A: Thank you. It was a typo (an additional “not”). We rephrased the sentence.
- page 9 row 36: citation is missing

A: We decided to exclude the mentioned sentence from the manuscript as it did not provide any meaningful contribution.

- page 9 row 42: not clear

A: We rephrased the sentence.
- discussion should be expand including other published papers, and in the same way other references should be added as for example Ozsaygili C, Duru N. Retina. 2020 Jun;40(6):1044-1052, Vadalà M, et al Clin Ophthalmol. 2020 Sep 15;14:2657-2665, Zur D, et al Acta Ophthalmol. 2020 Mar;98(2):e217-e223, Zur D, et al Ophthalmology. 2018 Feb;125(2):267-275..............
A: As you suggested, we improved the discussion section adding the appropriate references.

**Reviewer #4:** This is an interesting concept and OCT biomarkers predicting response to dexamethasone implants in DME is worthy of study. Unfortunately diabetic maculopathy, especially advanced or treatment resistant disease which is often treated with dexamethaonse implants is a chronic disease. Markers predicting response at 1 and 4 months is not as useful as longer term data and if you extended the study the results could be much more useful.
A: Thank you for evaluating out manuscript. We acknowledge the value of longer-term data as suggested by the reviewer. However, we believe that assessing the early results and examining the predictive value of OCT biomarkers during the initial 1 and 4 months of treatment response to dexamethasone implants in DME can also be of interest. Our analysis, focusing on ETDRS line improvement, could provide valuable data for clinicians to understand the contribution of various biomarkers to functional improvement in the early stages of treatment. We are essentially conveying that in the early stages, the only significant biomarker is central subfield thickness (CST). Therefore, clinicians would not need to evaluate an extensive array of other biomarkers to comprehend the initial visual recovery of patients.

The first/main conclusion (first sentence of conclusion) is that this study confirms the safety and efficacy of dexamethasone implants - this has already been clearly established in the literature and wasn't part of the aim/design of the study (particularly given there are only 33 eyes included making it hard to confirm the safety of a treatment)
A: Thank you for raising this point. We removed the sentence from the discussion and rephrased the first lines of the discussion.
I think there is some confusion in the discussion. You have stated on page 10 line 33 that dexamtethasone implants may not be considered first line especially in pseudophakic patients - It would be more likely to consider as first line in pseudophakic patients than phakic patients (though it is not typically considered first line in any patients).

A: Thank you for bringing up this crucial point. Indeed, it was a typo, and we have rectified it (it was also raised by reviewer 3).
